# Impact of Low Back Pain Clinical Trials Measured by the Altmetric Score: Cross-Sectional Study

**DOI:** 10.2196/jmir.9368

**Published:** 2018-04-05

**Authors:** Amanda Costa Araujo, Dafne Port Nascimento, Gabrielle Zoldan Gonzalez, Christopher G Maher, Leonardo Oliveira Pena Costa

**Affiliations:** ^1^ Masters and Doctoral Programs in Physical Therapy Universidade Cidade de São Paulo São Paulo Brazil; ^2^ Musculoskeletal Health Sydney School of Public Health The University of Sydney Sydney Australia

**Keywords:** Altmetric, social impact, clinical trials, low back pain

## Abstract

**Background:**

There is interest from authors and publishers in sharing the results of their studies over the Internet in order to increase their readership. In this way, articles tend to be discussed and the impact of these articles tends to be increased. In order to measure this type of impact, a new score (named Altmetric) was created. Altmetric aims to understand the individual impact of each article through the attention attracted online.

**Objective:**

The primary objective of this study was to analyze potential factors related with the publishing journal and the publishing trial that could be associated with Altmetric scores on a random sample of low back pain randomized controlled trials (RCTs). The secondary objective of this study was to describe the characteristics of these trials and their Altmetric scores.

**Methods:**

We searched for all low back pain RCTs indexed on the Physiotherapy Evidence Database (PEDro; www.pedro.org.au) published between 2010 and 2015. A total of 200 articles were randomly selected, and we extracted data related to the publishing trial, the publishing journal, methodological quality of the trials (measured by the 0-10 item PEDro scale), and total and individual scores of Altmetric mentioned and Altmetric reader. The study was a cross-sectional study, and multivariate regression models and descriptive statistics were used.

**Results:**

A total of four variables were associated with Altmetric mentioned score: impact factor (β-coefficient=3.4 points), number of years since publication (β-coefficient=–4.9 points), number of citations divided by years since publication (β-coefficient=5.2 points), and descriptive title (β-coefficient=–29.4 points). Only one independent variable was associated with Altmetric reader score: number of citations divided by years since publication (β-coefficient=10.1 points, 95% CI 7.74-12.46). We also found that the majority of articles were published in English, with a descriptive title, and published in open access journals endorsing the Consolidated Standards of Reporting Trials (CONSORT) statement.

**Conclusions:**

Researchers should preferably select high impact factor journals for submission and use declarative or interrogative titles, as these factors are likely to increase the visibility of their studies in social media.

## Introduction

There is growing interest from both authors and publishers in sharing the results of their studies over the Internet in order to increase their readership [1,2]. Similarly, consumers of research (including clinicians and patients) also share articles that they found interesting, useful, or controversial with their peers over the Internet. One of the ways that articles can be disseminated on a large scale is by sharing them on social media, such as Facebook, Twitter, Instagram, and others. Another way involves the use of reference manager websites such as Mendeley or Connotea [2]. Both methods offer new approaches to access, read, and discuss research articles; consequently, the dissemination of these articles increases [2-4].

The most traditional way of quantifying the scientific impact of an article or a journal is through the number of citations in peer-reviewed journals [5]. However, indices related to the number of journal citations do not necessarily reflect a greater dissemination of the content of articles to clinicians and patients. Until very recently, the impact of scientific articles on social media and reference managers was not quantified. In order to measure this type of impact a new score (named *Altmetric*) was created [2,6].

*Altmetric* is a tool developed by a group of British researchers [7]. *Altmetric* aims to understand the individual impact of each article through the attention attracted online (eg, on social media and reference managers) [6]. The *Altmetric* attention score is composed by two independent scoring systems: the *Altmetric mentioned* score and the *Altmetric reader* score [7]. The *Altmetric mentioned* score for an article reflects how widely an article is mentioned in a range of media, including social media (eg, Facebook, Twitter), newspapers, encyclopedias (eg, Wikipedia), online platforms (eg, Faculty1000 and Publication Peer-Reviews), videos on YouTube, sites on questions and answers (eg, Q&A stack overflow), and *policy documents* or PDF documents available over the Internet *.* Each of these mentions receives different weights to reflect the relative reach of each source, and contributes to the total score. For example, each mention on Facebook counts as 0.25 points while a mention on Twitter counts as 1.0 point. The *Altmetric reader* score can be visualized by clicking the *Altmetric* “donut” symbol (ie, a visual representation of the Altmetric score) and summing the number of readers. This score is hidden in the donut. The second score is the *Altmetric reader* score which measures the impact on online reference managers such as *Mendeley, CiteULike* and *Connotea* [7] *.* This score has identical weights for all reference managers (ie, 1.0 point for each mention). Readers can easily identify the *Altmetric mentioned* in the websites of most journals by clicking on the *Altmetric* “donut.”

Most journal articles about *Altmetric* published to date are only introductory tutorials or editorials [2,4-6,8]. Smith et al [9] published a discussion paper about the importance of *Altmetric* in the field of health sciences that aimed to quantify the social impact of these articles. Patthi et al [10] published a systematic review that retrieved seven articles published between 2010 to 2016 in the dental area that aimed to analyze the correlations between journal citations measured by the Web of Science website and *Altmetric mentioned* scores. This review concluded that journal citations and *Altmetric* scores are positively correlated (between r=0.30 and 0.61) [10]. Finally, Rosenkrantz et al [11] also observed positive correlations between citations and *Altmetric* scores in radiology journals.

There are three articles [12-14] that have measured the correlation among *Altmetric*, Tweets, blogs, and Mendeley (a reference management software). These studies observed a large increase in the number of Tweets and blogs related to scientific journals, and these variables were correlated with the *Altmetric* score *.* Previously published articles [13,15,16] showed that the number of Tweets can predict citations within the first three days of article publication. These findings indicate that *Altmetric* scores are likely to be correlated with the journal’s impact factor [8]. Rinald [17] published a tutorial about the benefits of open access journals with regards to visibility on social media. However, more research is needed to identify potential variables that might be associated with *Altmetric* scores, such as the journal impact factor, number of years since publication, study quality, and open access articles.

To our knowledge, there is no study describing the characteristics of randomized controlled trials (RCTs) and their *Altmetric* scores or predictive factors of *Altmetric* score. In this study, trials of nonpharmacological interventions for low back pain were chosen by the authors because back pain has the largest amount of evidence in the field of musculoskeletal health [18]. Additionally, back pain is extremely prevalent [19-21] and involves high costs [19,20,22]. According to a study that ranks the most disabling diseases in the world [21], low back pain has been one of the highest ranking musculoskeletal diseases since 1990 [21].

Therefore, the primary objective was to analyze potential factors related to the publishing journal (eg, online access) and the publishing trial (eg, trial quality) that could be associated with *Altmetric* scores in a random sample of low back pain RCTs. The secondary objective of this study was to describe the characteristics of these trials and their *Altmetric* scores.

## Methods

### Study Design

This is a cross-sectional study.

### Search Strategies

We selected a random sample of 200 low back pain RCTs from the Physiotherapy Evidence Database (PEDro) [23]. We have chosen PEDro because this is the most comprehensive database of physiotherapy trials [24,25], and also because the PEDro scale has acceptably high reliability and validity [18]. In addition, all trials indexed on PEDro are rated for methodological quality using the 0-to-10-point PEDro scale [24-27]. The items are described below:

1. Eligibility criteria
2. Random allocation
3. Concealed allocation
4. Baseline comparability
5. Blinding of subjects
6. Blinding of therapists
7. Blinding of outcome assessors
8. Completeness of follow up
9. Intention to treat analysis
10. Between-group statistical comparisons
11. Presentation of point measures and measures of variability

**Data sources and details that were extracted.**

- Downloaded from PEDro database: full title, authors’ names, journal name, language of publication, year of publication, category of intervention according to the PEDro database (ie, exercise, manual therapy, behavioral modification, electrotherapy, acupuncture), and PEDro total and individual scores.
- Extracted from the full-text article: continent where the study was conducted, type of title categorized as declarative (title expressing the results of the trial), interrogative (title introducing the trial in the form of a question), or descriptive (title describing the aim, but does not reveal the main conclusions).
- Extracted from Web of Science: journal’s impact factor, number of citations.
- Extracted from journal websites and *Directory of Open Access Journals*: if the paper was published as open access *.*
- Extracted from journal and Consolidated Standards of Reporting Trials (CONSORT) websites: if the journal endorses the CONSORT statement [28].
- Extracted scores from *Altmetric* website: total and individual scores of *Altmetric mentions* (individual scores [weights] from *Facebook [0.25 points]*, *Twitter [1.0 point], Google+user [1.0 point]*, *News Outlet [8.0 points]*, *Blogs [5.0 points]*, *Sina Weibo [1.0 point]*, *Reddit [0.25 points]*, *Linkedin [1.0 point], Highlight Platform [1.0 point]*, *Pinterest [0.25 points]*, *Wikipedia Page [3.0 points], Faculty1000 [1.0 point], Publication Peer-Reviews [1.0 point], YouTube [0.25 points], Q 0.25 points] &A [stack overflow; 0.25 points]* and *Policy Documents [3.0 points]*. We also collected total and individual scores related to *Altmetric reader* (individual scores from *Mendeley [1.0 point], CiteULike [1.0 point] and Connotea [1.0 point]).*

The total PEDro score is computed by summing *yes* responses to items 2-11. The first item does not count in the final score because this is related to external validity. All trials on PEDro are rated by at least two trained raters and, in cases of disagreement, a final arbitration is performed by a senior rater.

On February 1, 2016 we identified all low back pain trials indexed on PEDro that were published in the time period of 2010-2015 and selected a random sample of 40%. We excluded trial protocols, preliminary analyses of trials, and secondary analyses. The search strategy is described as follows:

Strategy search: “2010 until 2015” [year of publication] and “low back pain” [part of body] and “pain” [problem] and “clinical trial” [method].

### Data Extraction

Several pieces of data were extracted, as detailed in Textbox 1.

Data related to *Altmetric* scores and number of citations divided by years since publication were collected on May 10, 2016 for all articles because these scores are extremely dynamic.

### Statistical Analyses

The number of years since publication of the article and the number of citations were determined as of May 10, 2016. The number of citations was normalized by the number of years since publication (number of citations divided by years since publication), as it is expected that older manuscripts are more likely to have a larger number of citations compared to newer ones. Descriptive statistics were used to present most of the data.

Separate multivariate regression models were built to predict (1) *Altmetric mentioned* score and (2) *Altmetric reader* score. The independent variables in both models were: impact factor, paper was published as open access (yes/no), total PEDro score, number of years since publication, normalized citation count, and type of title. These variables were chosen because it seems plausible that they would be associated with *Altmetric* scores. For example, we choose the variable number of years since publication and total PEDro score because they are related to the number of accesses on PEDro [18].

Initially, univariate regression analyses were performed and all variables that reached a *P* value of <0.20 were retained for inclusion in the multivariate model. Multivariate regression models were then built and the final model contained only variables that reached a statistical significance of *P*<0.05. The results were expressed as R² indexes (explained variability of the model) and the individual contribution of each variable was expressed through the presentation of β-coefficients and their respective 95% CIs. We used the Statistical Package for Social Sciences (SPSS) version 19 for the analyses.

## Results

### Selection of Eligible Articles

**Figure 1:**
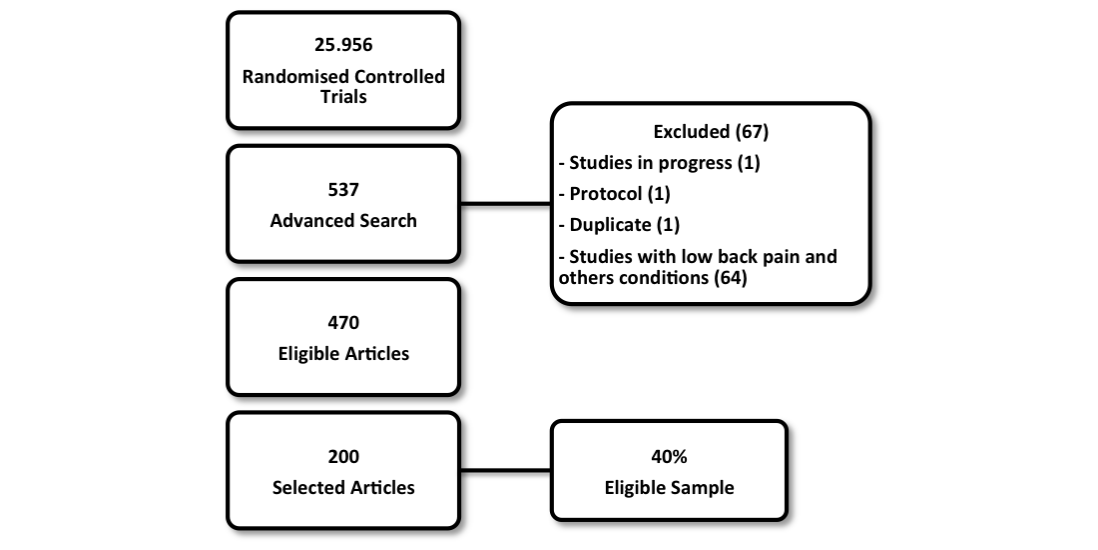
Flow diagram.

A total of 537 clinical trials were retrieved using the search strategy. Sixty-seven articles were excluded because they were related to conditions other than low back pain or were related to studies in progress. From the remaining 470 articles, 200 were randomly selected for analyses (Figure 1).

### Descriptive Characteristics of Articles

Table 1 presents the characteristics of the trials. From the 200 articles, 186 had an *Altmetric* score with a mean *mentioned* score of 18.2 (SD 41.3) and a mean *reader* score of 34.9 (SD 41.6). Most of the articles were published in English, had a descriptive title (title describing the aim, but does not reveal the main conclusions) and were published as open access in journals that endorse the CONSORT statement. In addition, the mean impact factor of the journals publishing these trials was 2.1 (SD 2.6) with a mean total PEDro score of 5.8 points (SD 1.6; Table 1).

### Predictive Factors

The univariate analysis for *Altmetric mentioned* score showed that being published in an open access journal was not independently associated with *Altmetric mentioned* score (Table 2). The final multivariate model is presented in Table 3. Four variables were associated with *Altmetric mentioned* score: impact factor (β-coefficient=3.4 points), number of years since publication (β coefficient=–4.9), number of citations divided by years since publication (β-coefficient=5.2 points), and descriptive title (β-coefficient=–29.4 points). This model accounts for 28% of the explained variance. The interpretation of this model is that older articles and those with descriptive titles were associated with a lower *Altmetric mentioned* score, whereas articles from journals with a higher impact factor and with greater citations were associated with a higher *Altmetric mentioned* score.

The univariate analysis for *Altmetric reader* score showed that being published in an open access journal was not associated with *Altmetric reader* score (Table 4). The multivariate analysis showed that one independent variable was associated with *Altmetric reader* score: number of citations divided by years since publication (β-coefficient=10.1 points, 95% CI 7.74-12.46). This single variable accounted for 31% of the explained variance.

**Table 1:** Characteristics of the included trails (n=200), *Altmetric mentioned* and *Altmetric reader scores*. Categorical data were expressed as numbers (percentage). Continuous normal data were expressed as means (SD). PEDro: Physiotherapy Evidence Database.

Variables		All articles (n=200)	*Altmetric mentioned*^a^ score (n=186)	*Altmetric reader* score (n=186)
Published in English (%)		198 (99.0)	18.4 (41.4)	35.3 (41.7)
Published in other languages (%)		2 (1.0)	0.0 (0.0)	0.0 (0.0)
**Continent where the trial was conducted (%)**				
	Asia	65 (32.5)	3.9 (5.1)	18.8 (25.3)
	Europe	70 (35.0)	13.6 (27.7)	40.0 (48.4)
	America	46 (23.0)	35.9 (59.8)	40.3 (39.8)
	Oceania	11 (5.5)	32.0 (35.9)	63.7 (49.3)
	Africa	8 (4.0)	34.2 (91.2)	31.1 (42.7)
**Category of interventions (%)**				
	Stretching, mobilization, manipulation and/or massage	88 (44.0)	28.5 (57.4)	42.8 (51.4)
	Strength training	85 (42.5)	17.6 (44.8)	31.6 (36.6)
	Behaviour modification	20 (10.0)	19.9 (43.0)	50.4 (64.3)
	Neurodevelopmental therapy, neurofacilitation	1 (0.5)	0.0 (0.0)	0.0 (0.0)
	Electrotherapies	24 (12.0)	11.3 (37.5)	10.0 (13.0)
	Acupuncture	13 (6.5)	11.5 (12.8)	20.0 (15.9)
	Skill training	41 (20.5)	26.6 (42.2)	51.5 (62.6)
	Education	50 (25.0)	16.1 (31.5)	35.0 (36.0)
	Fitness training	26 (13.0)	5.0 (14.1)	29.5 (35.5)
	No appropriate value	2 (1.0)	12.5 (7.8)	17.0 (7.1)
	Hidrotherapy, balneotherapy	8 (4.0)	3.4 (5.3)	17.1 (21.4)
	Orthoses, taping, splinting	1 (0.5)	16.2 (32.3)	52.8 (55.7)
**Type of title (%)**				
	Descriptive	180 (90.0)	15.2 (36.9)	32.4 (40.7)
	Interrogative/Declarative	20 (10.0)	43.6 (62.9)	56.0 (44.6)
**Open access (%)**				
	Yes	115 (57.5)	17.9 (42.8)	36.7 (44.0)
	No	85 (42.5)	18.6 (39.0)	32.3 (38.0)
**Journal endorses CONSORT statement (%)**				
	Yes	111 (55.5)	24.3 (50.1)	43.3 (47.5)
	No	89 (44.5)	9.2 (19.6)	22.5 (26.7)
**PEDro items (%)**				
	Eligibility criteria	166 (83.0)	19.4 (43.8)	37.7 (44.2)
	Random allocation	197 (98.5)	18.4 (41.4)	35.2 (41.8)
	Concealed allocation	96 (48.0)	25.3 (52.2)	42.3 (44.3)
	Baseline comparability	172 (86.0)	19.0 (43.4)	36.6 (43.4)
	Blinding of subjects	14 (7.0)	48.6 (76.3)	38.3 (39.2)
	Blinding of therapists	3 (1.5)	10.0 (10.5)	34.7 (30.8)
	Blinding of outcome assessors	78 (39.0)	27.3 (58.0)	42.9 (51.2)
	Completeness of follow up	129 (64.5)	20.8 (47.4)	39.0 (46.4)
	Intention to treat analysis	89 (44.5)	26.2 (46.7)	45.1 (42.0)
	Between-group statistical comparisons	195 (97.5)	18.5 (41.6)	35.5 (41.9)
	Presentation of point measures and measures of variability	187 (93.5)	17.9 (40.9)	35.2 (41.5)
Total PEDro score (mean [SD])		5.8 (1.6)			
Journal impact factor (mean [SD])		2.1 (2.6)			
Number of years since publication (mean [SD])		3.4 (1.7)			
Normalized citation count^b^ (mean [SD])		2.3 (2.3)			
**Score *Altmetric mentioned* (mean [SD])**				
	Tweeters	13.4 (31.0)		
	Facebook pages	3.9 (13.3)		
	Google+user	0.2 (1.0)		
	News outlet	0.3 (1.6)		
	Others	0.0 (0.0)		
	Total	18.2 (41.3)		
**Score *Altmetric reader* (mean [SD])**				
	Mendeley	34.4 (41.4)		
	CiteULike	0.5 (4.8)		
	Connotea	0.0 (0.2)		
	Total	34.9 (41.6)		

^a^Others are *Altmetric mentioned* by: Wikipedia page, Blog, Weibo users, Highlight platform, Policy documents, Post-publication peer-reviews, Linkedin, Reddit, Faculty1000, Q&A (stack overflow), Youtube, Pinterest.

^b^Normalized citation count calculated by number of citations divided by years since publication. Years since publication calculated by current year–year of publication.

**Table 2:** Univariate model to predict characteristics that were associated with Altmetric mentioned score. PEDro: Physiotherapy Evidence Database.

Variable		Constant	β-coefficient	95% CI	*P* value
**Journal characteristics**					
	Open Access	18.63	–0.65	–12.86 to 11.55	.92
	Impact Factor	10.32	4.23	1.95 to 6.52	.00
**Article characteristics**					.01
	Total PEDro score (/10)	–20.36	6.54	2.82 to 10.27	.00
	Number of years since publication	28.41	–3.01	–6.53 to 0.50	.09
	Normalized citation count (number of citations divided by years since publication)	3.61	6.11	3.84 to 8.38	.00
	Descriptive title	43.65	–28.47	–47.35 to –9.60	.00

**Table 3:** Final multivariate model to predict characteristics that were associated with Altmetric mentioned score.

Variable		Constant	β-coefficient	95% CI	*P* value
**Journal characteristics**		43.02		22.42 to 63.63	.00
	Impact Factor		3.42	0.98 to 5.86	.00
**Article characteristics**					
	Number of years since publication		–4.99	–8.50 to –1.47	.00
	Normalized citation count (number of citations divided by years since publication)		5.18	2.49 to 7.88	.00
	Descriptive title		–29.36	–46.48 to –12.23	.00

**Table 4:** Univariate model to predict characteristics that were associated with Altmetric reader score. PEDro: Physiotherapy Evidence Database.

Variable		Constant		β-coefficient		95% CI		*P* value	
**Journal characteristics**									
	Open Access		32.33		4.34		–7.96 to 16.64		.49
	Impact Factor		27.53		4.83		2.25 to 7.40		.00
**Article characteristics**									
	Total PEDro score (/10)		–8.10		7.29		3.56 to 11.03		.00
	Number of years since publication		23.59		3.36		–0.19 to 6.90		.06
	Normalized citation count (number of citations divided by years since publication)		11.45		10.10		7.74 to 12.46		.00
	Descriptive title		56.05		–23.67		–42.87 to –4.47		.02

## Discussion

### Principal Findings

The primary objective of this study was to analyze potential factors that could be associated with *Altmetric* score. The secondary objective was to describe the characteristics of low back pain RCTs and their *Altmetric* scores. We found that trials with interrogative/declarative titles, those published in higher impact factor journals, those published more recently, and those with a larger number of citations were associated with a higher *Altmetric mentioned* score. We observed that the number of citations was also associated with a higher *Altmetric reader* score. Finally, we found that the *Altmetric reader* score was higher than *Altmetric mentioned* score. Most of the articles were published in English, had descriptive titles, and were published as open access in journals that endorse the CONSORT statement.

There are three previous articles that have measured correlations between the number of citations and *Altmetric* scores in medical journals [10,11,29], and numerous others studying the relationship between tweets (the main *Altmetric* score component) and citations ([15] being the first). The conclusions of these articles are very similar to our study: there is an association between citations and *Altmetric* scores. This information confirms that conventional measures of scientific impact (based on citations) are associated with social impact (based on social media). The difference between our study and these three previous studies is that we used multivariate regression analyses rather than simple correlations. We believe that this approach allowed us to focus on the key independent variables, and the beta coefficients we provide are more interpretable than correlation coefficients. For example, we can predict that for every citation received, 5.2 and 10.1 points will be added to the *Altmetric mentioned* and *reader* scores, respectively. Scientific impact appears to follow the social impact in back pain trials.

We observed an association between *Altmetric mentioned* score and the journal’s impact factor. The journal’s impact factor is a measure that reflects the number of citations of scientific articles published in the journal divided for the two previous years [5]. We might infer that journals with higher impact factors have more credibility to a wider range of readers, health care providers, and media, which may reflect a large number of posts in social media. Many of these journals may have well-developed media strategies, such as preparation and distribution of releases to the media. This action encourages the promotion of their papers in newspapers, blogs, and social media more rapidly and efficiently than journals that do not do this. These journals also format the online versions of articles so that a reader can easily click an icon to post details about the study on social media, usually by embedding a key figure from the article in the post.

We also observed that trials with declarative/interrogative titles were associated with higher *Altmetric mentioned* scores than those with descriptive titles. Our study is, to our knowledge, the first that has investigated the effect of the format of the articles title on *Altmetric* scores. There is evidence that articles with shorter titles are more likely to be highly cited [30-32]. Jamali et al [33] concluded that articles with interrogative titles are also associated with a larger number of citations and downloads [33]. Therefore, authors should be aware that shorter and interrogative titles should be considered in order to attract a wider audience for their manuscripts. Finally, we observed that papers published more recently also have a higher *Altmetric* score; it seems that recent studies are more likely to be shared. This finding should be investigated further in future studies.

The strength of this study is the use of a representative sample of trials (N=200, or 40% of all trial reports indexed on PEDro and categorized as “low back pain”). A possible limitation of this study is related to external validity, as our dataset contains only articles about low back pain. It would be important to replicate our study in other health disciplines.

### Conclusion

Our study brings new insights for authors on how to increase the visibility of their articles. First, researchers should preferably select high impact factor journals for submission and use declarative or interrogative titles, as these factors are likely to increase the visibility of their studies in social media. Furthermore, we suggest new studies that use different research designs (eg, systematic reviews and guidelines) in order to externally validate our findings.

## Abbreviations

CONSORT: Consolidated Standards of Reporting Trials
PEDro: Physiotherapy Evidence Database
RCT: randomized controlled trial
SD: standard deviation
